# Making the most of bioimaging data through interdisciplinary interactions

**DOI:** 10.1242/jcs.262139

**Published:** 2024-10-23

**Authors:** Virginie Uhlmann, Matthew Hartley, Josh Moore, Erin Weisbart, Assaf Zaritsky

**Affiliations:** ^1^ European Bioinformatics Institute (EMBL-EBI), EMBL, Cambridge CB10 1SD, UK; ^2^ BioVisionCenter, University of Zurich, Zurich 8057, Switzerland; ^3^ German BioImaging-Gesellschaft für Mikroskopie und Bildanalyse e.V., Constance 78464, Germany; ^4^ Imaging Platform, Broad Institute of MIT and Harvard, Cambridge, MA 02142, USA; ^5^ Department of Software and Information Systems Engineering, Ben-Gurion University of the Negev, Be'er-Sheva 8410501, Israel

**Keywords:** Bioimage analysis, Bioimaging, Interdisciplinarity, Microscopy, Open science

## Abstract

The increasing technical complexity of all aspects involving bioimages, ranging from their acquisition to their analysis, has led to a diversification in the expertise of scientists engaged at the different stages of the discovery process. Although this diversity of profiles comes with the major challenge of establishing fruitful interdisciplinary collaboration, such collaboration also offers a superb opportunity for scientific discovery. In this Perspective, we review the different actors within the bioimaging research universe and identify the primary obstacles that hinder their interactions. We advocate that data sharing, which lies at the heart of innovation, is finally within reach after decades of being viewed as next to impossible in bioimaging. Building on recent community efforts, we propose actions to consolidate the development of a truly interdisciplinary bioimaging culture based on open data exchange and highlight the promising outlook of bioimaging as an example of multidisciplinary scientific endeavour.

## Teaser

**Summary:** A proposal for actions needed to consolidate the development of an inclusive and open culture of data sharing in bioimaging and a highlight of the promising outlook of multidisciplinary scientific collaborations.

## Introduction

Over the past decades, imaging technologies have undergone significant advancements, enabling the routine acquisition of terabytes of data from biological experiments that span a broad spectrum of spatial and temporal scales (Abraham, 2004; Pepperkok and Ellenberg, 2006; Ouyang and Zimmer, 2017). Concurrently, technological innovations are continually expanding the boundaries of resolution and sample volumes, pushing the capabilities of imaging to unprecedented levels. These include developments in light microscopy that go beyond the diffraction limit and generate super-resolution images (Lelek et al., 2021) or that allow dynamic 3D imaging over a wide range of scales (Chen et al., 2014; Gwosch et al., 2020). Equally exciting progress has propelled volumetric electron microscopy techniques to the forefront of life science research (Collinson et al., 2023). The development of an ever-wider range of imaging protocols and probes allows for the acquisition of information-dense images that reveal the spatial location and dynamics of intracellular components (Xia et al., 2019; Frei et al., 2022). Protocols combining multiple imaging modalities are emerging as well (Bischof et al., 2024) – with spatial transcriptomics (Marx, 2021) and correlative light-electron microscopy (CLEM) (de Boer et al., 2015) being particularly notable examples.

Fortunately, our capacity to quantitatively analyse bioimages has expanded in tandem with our ability to acquire them. Modern machine learning and artificial intelligence has been transformative in that area, and has led to the development of innovative methods that offer unprecedented automation capabilities to address problems, such as image restoration (Weigert et al., 2018), nuclei and cell detection (Schmidt et al., 2018; Hung et al., 2020) and segmentation and tracking of complex cellular and subcellular structures (Heinrich et al., 2021; Isensee et al., 2021; Stringer et al., 2021; Lefebvre et al., 2024 preprint). Although these technological advancements provide a boon for life science research, they carry associated challenges. Most significantly, the management and storage of vast image datasets along with quantitative analysis at scale pose significant computational hurdles that necessitate the creation of sophisticated cyberinfrastructure and tools (Allan et al., 2012; Eliceiri et al., 2012; Moore et al., 2021; Marconato et al., 2024). A second challenge is ensuring reproducibility of bioimage analysis and methods to maximise potential reuse. The ‘FAIR’ guiding principles for scientific data management and stewardship – findability, accessibility, interoperability and reusability (Wilkinson et al., 2016) – can and should be applied to digital entities attached to them, such as metadata and analysis infrastructures.

The increasing technical complexity of all aspects involving bioimages, ranging from their acquisition to their analysis, has led to a diversification in the expertise of scientists engaged at the different stages of the discovery process. Individuals working with bioimages now come from varied technical disciplines, belong to distinct communities, communicate in unique domain-specific terminologies and have different interests (Jamali et al., 2022). This diversity of profiles poses a serious challenge – establishing fruitful methods of communication and interaction between individuals with the skills to generate data and those with the skills to analyse it. However, the potential reward of these interactions matches the scale of the challenge, as it presents an exceptional opportunity for authentic interdisciplinary scientific collaborations. In this Perspective, we illustrate the various types of profiles populating the bioimaging research universe and identify the primary obstacles that currently hinder their interactions. We advocate that data sharing is the cornerstone of modern bioimaging and lies at the heart of innovation within this community. After decades of data sharing in bioimaging being viewed as next to impossible, the readiness and willingness to share bioimaging data has shown remarkable growth over the past 10 years (Zaritsky, 2018). Building upon the recently published community efforts to formulate requirements for appropriate bioimaging cyberinfrastructure (Bajcsy et al., 2024 preprint), and to produce well-documented, high-quality and ready-to-share image data (Bialy et al., 2024 preprint), we propose first steps towards the implementation of a truly interdisciplinary culture based on open data exchange. We highlight ongoing community efforts, put forward ideas of actions to further consolidate the community and discuss the promising outlook of bioimaging as a truly multidisciplinary scientific endeavour.

## The bioimaging research ecosystem

### Data producers and data consumers

Bioimaging is inherently an interdisciplinary field populated with scientists from highly diverse scientific backgrounds. Despite their diversity, it is possible to broadly categorize these individuals into two main groups based on their primary area of expertise: those involved with generating data and those concerned with utilizing data produced by others. We refer to these two groups as ‘data producers’ and ‘data consumers’, respectively. We deliberately use this terminology instead of academic job categories because, as we shall discuss, a researcher might equally be a producer or a consumer depending on the situation.

Data producers are typically experimentalists who are acquiring data to answer a specific biological question or to create a new resource. This data can range in terms of scale from a couple of images to gigantic datasets designed to leverage statistical power, and in terms of novelty from a standard protocol for a commercial microscope to novel approaches for a custom system. Data producers can also range in scope from individuals to large research consortia (Regev et al., 2017; HuBMAP Consortium et al., 2019; Sunagawa et al., 2020). Although they are often life scientists, data producers can also be facility staff, optical engineers, physicists who generate images to calibrate microscopy systems or to showcase the capabilities of a new imaging technology, or chemists acquiring images to demonstrate proof-of-concept for novel imaging reagents such as fluorescent probes.

Data consumers are often computer scientists, mathematicians, physicists or engineers whose interest is in developing novel ways to analyse imaging data. Such method developers usually approach biological problems with a training background that differs from those in the life sciences. In order to validate and demonstrate the value of their theoretical or algorithmic contributions, method developers need imaging data on which to build, test and benchmark their new methods. Additional types of data consumers include computational cell biologists motivated by mining or reanalysing ‘old’ data in new ways to find new patterns and generate new biological insight and hypotheses, or scientists teaching biology or bioimage analysis.

The work of data consumers depends on the availability of image data generated by data producers, especially a sufficient amount of high-quality data along with its associated metadata; concurrently, the work of data producers is greatly enhanced by the development of analysis methods driven by data consumers. Additionally, producers can also be consumers at times. For example, experimental biologists might want to compare data acquired with a new setup or protocol to a reference dataset for quality assurance, attempt to reproduce published results before engaging in a new project, or seek to replicate published workflows to better understand them before adapting and applying them to their own data.

### Obstacles to interaction and communication

**Fig. 1.**
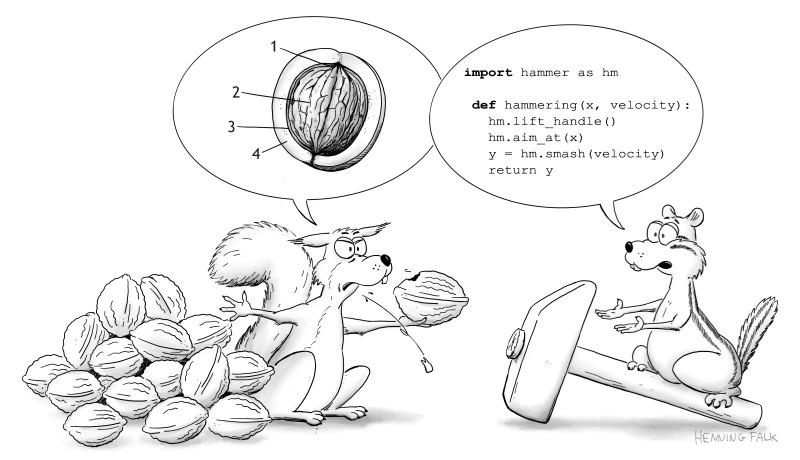
**Data producers and data consumers speak different languages.** Data producers (e.g. experimental biologists) and data consumers (e.g. computer scientists) approach problems from different perspectives. Although their respective domain-specific expertise is highly complementary, mutual understanding and communication is often challenging and hampers interdisciplinary collaboration. The cartoon is ‘Producers and Consumers’ by Henning Falk (doi:10.5281/zenodo.12751376), ©2024 NumFOCUS, where it was published under a CC BY 4.0 licence.

As the lifeblood of both producers and consumers is the image data itself, we advocate that the way towards bringing producers and consumers together relies on developing and nurturing a culture of open data sharing in the bioimaging community. However, mutual understanding and communication between producers and consumers, although necessary to tackle the most challenging questions in biology and move the field forwards, is often difficult (Fig. 1). Impediments in communication can arise from differences in technical backgrounds, interests, priorities and incentives. For instance, experimental data producers often prioritize quantifying their data to find a specific biological signal, whereas tool-developing data consumers might prioritize algorithmic novelty and availability of high-quality, annotated and curated data that can be reused. However, sufficient incentives for producers to share their data are lacking. Furthermore, because of the variety and complexity of biological imaging, maximizing the long-term value of image data, particularly in the context of reuse and integration, always entails extra work such as quality control, metadata documentation and formatting steps. This comes at a cost that data producers are understandably reluctant to pay, particularly when they might not have dedicated resources for this effort and might not see a direct benefit to it. Refusing to carry out this extra work nonetheless thwarts opportunities for data reuse and is likely to lead to a duplication of efforts.

As sharing image data costs both time and money, the question of what makes a particular dataset worth sharing is of central importance. Producers and consumers can have differing perspectives on what constitutes useful data. Arguably not all data need to be shared, such as data of low quality acquired in the preliminary setup of an experiment, or data for which supporting metadata have been lost and therefore cannot be given proper context. Conversely, data of seemingly little to no value to experimentalists might be a rich resource for consumers when accompanied with appropriate metadata. Microscopy calibration data offers one such example. Whereas producers might consider such data only necessary preparation for actual experiments, it may be a goldmine for consumers developing computational imaging methods. Although it is ultimately hard to predict whether image data will be useful to others, a successful culture of data sharing also relies on experimentalists actively considering whether and how others could use their images, a process that is greatly facilitated by direct interactions between data producers and consumers (Ellenberg et al., 2018).

Another consideration is that data consumption is also often separated temporally from data production−a novel analysis tool might be applied on datasets from years past, but only if these old datasets can be found, accessed and interpreted, which is only possible if metadata was kept along the way. This is best exemplified by the transformative AlphaFold model for protein structure prediction, whose remarkable performance is in large part due to the availability of an extensive amount of curated data publicly available in the Protein Data Bank (Jumper et al., 2021). The success of AlphaFold demonstrates that lowering the cost and time of data deposition, curation and storage using public platforms is a necessary first step to stimulate producers to share their image data. Going further, additional incentives should include stricter policies promoting open data sharing by scientific journals and funding agencies, as well as inclusion of the original data producer as an author in (re)analysis works.

### A growing interdisciplinary ecosystem

The challenges highlighted above have contributed to the ongoing development of an ecosystem of supporting players in bioimaging, who offer an unprecedented opportunity to overcome barriers to interactions between producers and consumers. New actors in the bioimaging research universe include data stewards (also referred to as curators), research software engineers and bioimage analysts, among others. Data stewards combine domain knowledge in biological imaging with experience and training in the practices of data management. By mediating the process of data sharing, they lower the barriers for producer–consumer interaction by assisting individual data producers with curating and depositing their datasets and executing data management plans. They also actively help producers by familiarizing them with the existing infrastructure for data sharing and teaching them how to prepare datasets in a way that can be reused by others, thereby mitigating some of the factors that cause reluctance in data sharing (von Suchodoletz et al., 2021; Kemmer et al., 2023).

Research software engineers combine professional software development skills with some understanding of biological research and provide expertize in the implementation, development and maintenance of software solutions. They contribute both by building solutions to help consumers locate and access data and by assisting producers in using data analysis methods. Bioimage analysts connect tool developers to data producers by matching the most appropriate bioimage analysis tools to the type of data required to solve a specific scientific question (Miura and Sladoje, 2020). They build analysis pipelines, train producers on how to use them and can propose experimental adjustments to make the data more suitable for analysis. Data stewards, software engineers and bioimage analysts are therefore poised to act as facilitators between producers and consumers and to enable the development of better communication around data sharing that will ultimately lead to richer science.

## Data sharing platforms are a fertile ground for interdisciplinary exchanges

Sharing bioimage data can serve as an initial connection point between producers and consumers. This might appear straightforward, but as discussed above, finding concrete ways to enable quick and effective sharing of data can be challenging. Large research initiatives that identified the benefit of data sharing early on have paved the way forward by setting up public resources of microscopy image data that have generated many positive returns. For example, data from OpenCell (https://opencell.czbiohub.org), the Human Protein Atlas (https://www.proteinatlas.org) and the Allen Cell Explorer (https://www.allencell.org) have been used to validate new methods to represent protein subcellular localization (Kobayashi et al., 2022). These representations were then used to create a Neuronal Organellomics Vision Atlas (Molitor et al., 2024 preprint). Similarly, data from OpenOrganelle (https://openorganelle.janelia.org) and the JUMP Consortium (Chandrasekaran et al., 2023 preprint) as well as similar datasets in the Cell Painting Gallery (Weisbart et al., 2024 preprint) and the Mitotic Cell Atlas (https://www.mitocheck.org/mitotic_cell_atlas) are driving the development of novel machine learning techniques for subcellular object segmentation (Conrad and Narayan, 2023; Xie et al., 2023), image-based phenotyping (Tegtmeyer et al., 2024; Tomkinson et al., 2024 preprint) and continuous cellular process modelling (Szkalisity et al., 2021). These examples, among many others, are excellent demonstrations of the impact that data sharing can have. However, the resources required to set up a public platform for image data hosting and sharing are significant, and although large project consortia might be able to gather such support, we should not assume that individual labs can afford to do so. Furthermore, expecting every institution or project to develop, populate and maintain their own public image repository would represent a significant duplication of effort.

Although the benefits of and the need for generalist bioimage data sharing platforms have been apparent for many years and were indeed recognized early on (Linkert et al., 2010), the intrinsic complexity of image data has slowed down efforts to provide generally open platforms. In structural biology, community archiving has centred around the Protein Data Bank (wwPDB consortium, 2019). Together with the advent of structure determination by cryo-EM, this gave rise in 2013 to the Electron Microscopy Public Image Archive (EMPIAR) (Iudin et al., 2023), a platform dedicated to sharing the raw images underlying electron density maps. This was followed in 2016 by the Image Data Resource (IDR) (Williams et al., 2017) for selected reference datasets in both light and 2D electron microscopy, and then in 2019 by the BioImage Archive (Hartley et al., 2022) as a general resource for routine life science image deposition. These resources form part of a larger ecosystem of bioimage data sharing platforms available to users, which include considerations for data type and levels of data specificity (Table 1).

**Table 1.**
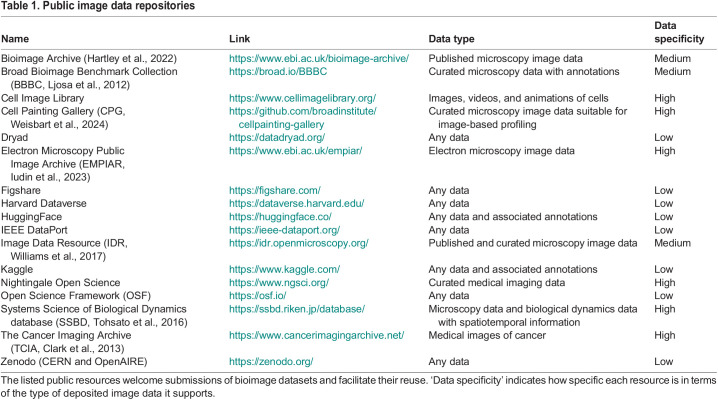
Public image data repositories

The bioimaging community is increasingly demonstrating the long-term benefits of sharing bioimage data through generalist data sharing platforms. One of the essential factors at the core of building these platforms is the standardization of file formats and metadata. Although this poses a major challenge for every type of data, standardization of microscopy images is at the extreme end of the difficulty spectrum as a result of the virtually endless possible combinations of sample preparation, imaging device, acquisition parameters, formats, dimensions and sizes. Efforts towards identifying minimum metadata standards have a long history in communities dealing with imaging modalities that inherently generate large datasets, such as high-content screening microscopy and multiplexed tissue imaging (Li et al., 2016; Schapiro et al., 2022; Hosseini et al., 2023). In recent years, the broader bioimaging community has been particularly active in proposing metadata standards (Sarkans et al., 2021) and guidelines (Zulueta-Coarasa et al., 2023 preprint) that are general enough to apply to all kinds of bioimages and that specifically aim to enable sharing and reuse by data consumers. Additionally, the recent introduction of the OME-Zarr format (Moore et al., 2023) is a major leap towards the last crucial element in the chain from data production to consumption – standardization of bioimage data file storage and handling. The dream of standardization for bioimaging data across the board is within closer reach than ever; however, much remains to be done to achieve a scientific culture of image data sharing. Improving the ease of finding suitably well-documented datasets is a particularly important goal.

## Datathons as a first step towards richer interactions between producers and consumers

Developing novel ways of working and boosting scientific discoveries in bioimaging is still impeded by difficulties in communication between the many actors along the producer–consumer spectrum, which include different approaches to communication of unpublished work, publication strategy and authorship attribution. Such obstacles can only be alleviated by open and transparent interactions between all parties involved (Schlaeppi et al., 2022). Significant resources have been invested into providing data platforms and defining roles that support their use. We are now at a point where more bioimage data sharing infrastructure is available than ever before, yet there remains a significant hurdle towards normalizing bioimage data sharing in the life sciences that is human rather than technical: developing a common language that will allow different communities in bioimaging to collaborate closely and efficiently. Such complex communication requires coordination, which is greatly facilitated by organizations that operate at local, national and international levels. Successful examples of networks actively promoting cross-disciplinary interactions in bioimaging include EuroBioImaging (Pfander et al., 2022), GlobalBioImaging (Swedlow et al., 2021), Bioimaging North America (BINA) (https://www.bioimagingnorthamerica.org), BioimagingUK (https://bioimaginguk.org), the German Bioimaging Society for Microscopy and Image Analysis (GerBI) (https://gerbi-gmb.de), the Global BioImage Analysts' Society (GloBIAS) (https://www.globias.org/), and the AI4Life project (https://ai4life.eurobioimaging.eu).

**Fig. 2.**
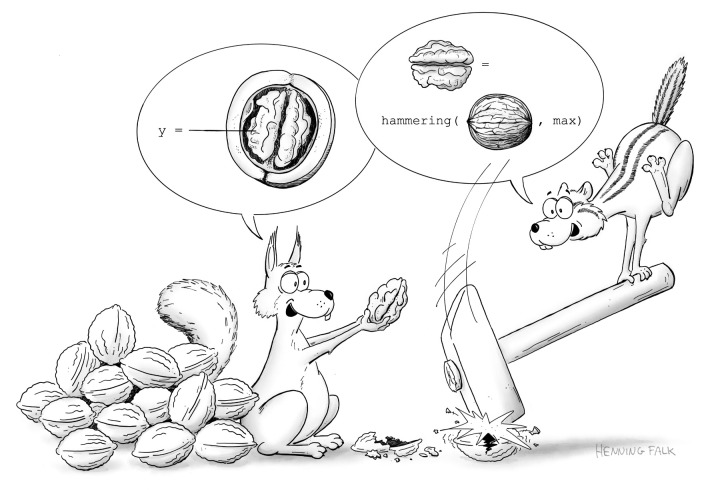
**Interdisciplinary collaboration is key to getting the most out of complex data.** Developing efficient ways to communicate (e.g. a common language) allows data producers and consumers to work closely together and ‘crack tough nuts’, that is, tackle challenges that would have been too hard for either to take on alone. The cartoon is ‘Common language’ by Henning Falk (doi:10.5281/zenodo.12751376), ©2024 NumFOCUS, where it was published under a CC BY 4.0 licence.

Although bioimaging networks carve out a discussion space that equally belongs to producers and consumers, a further step is necessary to trigger these two groups to go beyond simply communicating their own research to each other and start working hand-in-hand. We therefore propose use of ‘datathons’ to bring the producer and consumer communities together at a hands-on level (Aboab et al., 2016). Datathons take inspiration from ‘hackathons’ (i.e. social coding-focused events) but bring together all actors along the producer–consumer spectrum instead of regrouping participants with similar technical expertise. The potential of hackathons has already been successfully leveraged in the life sciences in the context of spatial data analysis (https://spatialhackathon.github.io/) and image analysis method benchmarking (i.e. assessing the performance of different methods against each other) (https://eubias.org/NEUBIAS/venue/neubias/benchmarking-sample-datasets-wg5/). For example, the recent AI4Life Open Calls provide a glimpse of the positive impact of focused interactions between scientists across the data producer-consumer spectrum (Box 1). By working together and finding efficient ways to communicate, producers and consumers are given the opportunity to ‘crack tough nuts’ together, that is, solve hard challenges that neither could tackle alone (Fig. 2).

**Box 1. The AI4Life Open Call – a success story of interdisciplinary collaboration in bioimaging**

The AI4Life project aims to provide research services and infrastructure that support the use and development of AI and machine learning in bioimage analysis. The AI4Life Open Calls (https://ai4life.eurobioimaging.eu/create-your-website-with-blocks/open-calls/) provide an excellent illustration of successful direct interactions between producers and consumers. Open Calls were designed to bring together computational experts from the AI4Life team and biologists who need help applying machine learning-based methods to their specific bioimage data. Proposals are evaluated following a comprehensive selection procedure involving a panel of international reviewers who score submissions based on overall quality, amount of effort required for successful completion and level of ‘enthusiasm’ elicited by the project. This latter point aims to capture whether the problem is exciting from the point of view of data consumers and is therefore essential to ensure that the collaboration is stimulating for all parties involved. Successful proposals receive direct support from the AI4Life team. The first Open Call was launched in 2023 and received an enthusiastic response, with a total of 72 applications from a wide range of areas (e.g. cancer research, marine biology, plant biology, ecology, microbiology and immunology), of which eight were selected. This success was repeated in the second Open Call in early 2024, which received 51 applications and introduced a two-phase process in which 20 selected applicants are given the opportunity to engage with experts for recommendations to guide their own analysis, before a smaller subset of projects is chosen to receive in-depth support. The AI4Life Open Calls have proved to be a rare opportunity for data producers and consumers to engage directly. This initiative can be seen as a first step towards the datathon format we propose, which would ultimately involve participants from a wider range of institutions on the data consumer side. Moving beyond consumers supporting producers through technical consultations, as in the AI4Life Open Calls, datathons would aim to foster longer-term collaborative work in which all parties are equally involved in the project design, encouraging the initiation of long-lasting scientific collaborations. For such endeavours, an in-person component will be crucial for facilitating the development of close interactions.

By supporting shared work on novel reanalysis of existing public data, dataset annotation and curation, datathons can serve two purposes. The first is to demonstrate the immediate scientific benefit of producer–consumer interactions, and the second is to develop the long-term links between these communities that will eventually normalize data sharing and reuse. Datathons would also offer an opportunity for representatives from established imaging resources to identify new uses for their data and offer precious insights on how to best support the needs of the community. For datathons to be maximally productive, bioimage data should be curated and prepared in advance of the meeting when possible. Public requests for open data, emulating a ‘most wanted’ board for bioimage datasets, echoed by a similar ‘most wanted’ board of public requests for analysis pipelines, could help to seed collaborations.

**Fig. 3.**
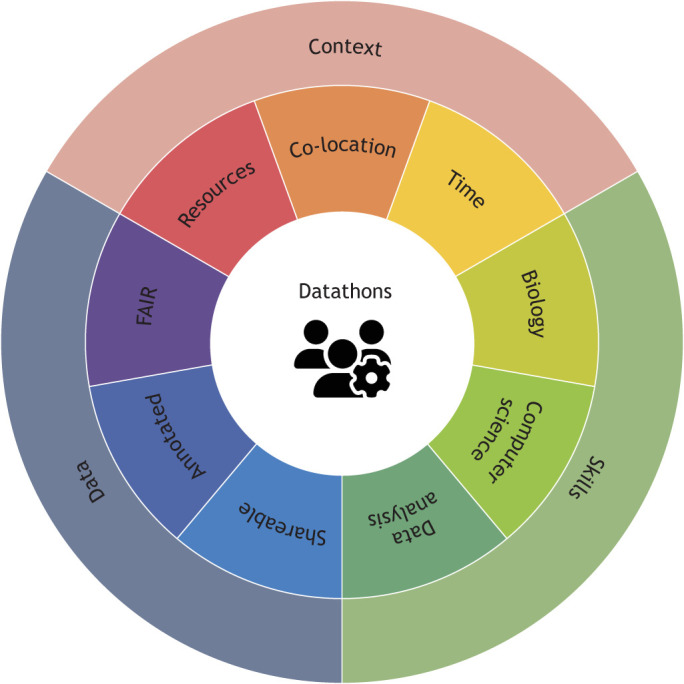
**Datathons combine the elements needed for productive interdisciplinary interactions.** By bringing together ready-to-(re)use bioimaging data, diverse scientific skills, and a shared context, datathons are a concrete action that can be implemented to push for a broader culture shift towards truly interdisciplinary collaborations in the bioimaging space. The icon ‘users-gear’ is from Font Awesome, where it was published under a CC-BY 4.0 licence.

Datathons are a solid first mechanism to kindle productive interdisciplinary interactions that leverage the strength and impact of the diverse scientific profiles evolving in bioimaging (Fig. 3) but cannot alone effect a broader culture shift away from distinct scientific communities. Firstly, all members of the community cannot realistically be physically present in the same room at the same time for economical or personal reasons. Complementary and inclusive solutions that ensure that all members of the community feel included in the discussions, invited to contribute and heard are thus needed to develop a culture of open but also distributed collaboration. This can begin, for example, by ensuring that the outcomes of datathons are published as open access reports to increase their visibility. Optimally, these could be published minimally as preprints following the BioHackrXiv template (https://guide.biohackrxiv.org/) established by the BioHackathon series (http://www.biohackathon.org/). In general, discussions on publicly available bioimage datasets should be open-by-default regardless of their degree of formality, emulating the culture of open dialogues about codebases that have been facilitated by the use of GitHub issues, which provide a way to transparently plan, discuss and track work in a collaborative manner. This can be achieved by guaranteeing that all stakeholders can freely sign up to join the discussions without needing permission from a higher administrator. A working example of this is the image.sc forum (Rueden et al., 2019), which enables open discussions between method developers and their users. The Image.sc LIVE around the world event (https://forum.image.sc/t/image-sc-live-around-the-world/89596), which invited participants to engage in live discussions with data analysis experts from around the world through a virtual chat, offers an example of how a delocalized datathon could be organized. Going forwards, if existing platforms do not offer an appropriate forum for the different actors in the bioimaging space to meet and interact, then new ones need to be created.

Besides the inherent challenges with communication across scientific fields, growing a culture of effective sharing and reuse of image data will require, like any other cultural change, a sustained effort over a long period of time. Funding for such events in the imaging community has lagged behind that for areas with a greater tradition of data sharing and reuse. In structural biology or genomics, for example, the European infrastructure for life sciences data (ELIXIR) regularly sponsors data hackathons (https://biohackathon-europe.org/). Once seeded through events such as datathons, a more profound culture change will also require sustained investment embedded in the research lifecycle (Mons, 2020) together with the actions of individuals, groups and institutions that stimulate and nurture interdisciplinary collaborative work. The same is naturally true for a culture of bioimaging data sharing if it is to stand the test of time. Building a long-term connection between scientific fields requires a commitment of resources and active effort toward bringing consumers and producers together. This need is not met by the current funding models, which encourage individual scientists on either end of the producer–consumer spectrum to favour novelty instead of investing time and energy into existing data and algorithms, leading to a culture that is the antithesis of the FAIR principles.

Finally, cultivating a culture of data sharing will likely require changes to the research credit system that both incentivize and track data reuse. Ideas to achieve this could include requiring publications to identify when publicly available data have been used. Such a policy might involve capturing information on the source of the reused data, the original author and possibly on how the public dataset was identified. Recording this information would first and foremost allow a better understanding of the current state of data reuse in the life sciences and identify the biggest points of frustration or resistance associated with identifying or crediting public datasets. This would then also provide a valuable method to assess the extent and benefit of data reuse, which would in turn generate more sharing incentives by helping producers appreciate the value of making their data publicly available. Ideally, the bioimaging community should collectively explore such ideas to identify the best creative solutions.

## Tackling the bioimaging data challenge together

As biology is at its roots an observational science, bioimage data is at the heart of our understanding of living systems. Pushing the boundaries of this understanding, however, requires a wealth of different expertise. As such, data sharing is key to reaching the necessary experts, enabling much more than individual collaborations, and contributing to a deeper culture shift away from isolated scientific disciplines and towards interdisciplinary research. Dedicated training programmes covering the many different aspects of bioimaging, from data acquisition to management, analysis and quality control, will be required for more frequent and productive interdisciplinary collaborations (Box 2).

**Box 2. Training the interdisciplinary scientists of tomorrow**

The needs arising from computational analysis of bioimaging data will also require the creation of interdisciplinary curricula that equip scientists with the skills needed to be both producer and consumer of their own data from early on in their career. The development of formal bioimage analysis-focused programmes to meet the growing demand for bioimage analysts in academia and industry will be incredibly valuable to promoting a culture of bioimage data sharing. Example of such curricula include the Advanced Microscopy Postdoctoral Fellowship Program started in 2013 at Harvard Medical School, a pioneering course of study combining a component of independent research in microscopy with training in the technical, teaching and administrative skills needed to support scientific research in the context of a core facility (Waters, 2020). Across Europe, the Training Schools organised by the Network of European BioImage Analysts (NEUBIAS) from 2016 to 2020 have revealed the immense need for training at all career stages, and the necessity of a forum to allow the exchange of knowledge and experience between different communities of experts (Martins et al., 2021). The success of the Training Schools inspired the NEUBIAS Academy (https://neubiasacademy.org/), an initiative to develop a community-driven framework for training in bioimage analysis. In 2019, the first ever Bioimage Analysis Postdoctoral Training Programme was launched at the Broad Institute. In just under five years of operation, fellows enrolled in this programme have demonstrated an exceptional level of collaborative engagement, leading to impressive amounts of research outputs (Cimini et al., 2024). Its main drivers of success have been the excellent communication and organizational skills of the participants. These successful examples are raising interest and awareness for interdisciplinary training that blurs the lines between data producers and consumers. Such efforts will certainly contribute to new higher education curricula covering the whole bioimaging data life cycle, from acquisition to analysis, that can have a strong impact in shaping future scientists to be more mindful of the importance of bioimaging data management and sharing.

The opportunities for biological research offered by cutting-edge artificial intelligence have already provided a sneak peek into what lies ahead and invite speculation as to yet unknown possibilities that might arise out of close interactions between those generating data and those mining information out of them (Nogare et al., 2023). Communication between data producers and consumers is now possible more so than ever, thanks to community agreements on metadata, standardized file formats and training programmes that provide early exposure to a wide range of disciplines. Beyond consensus on these specifications, however, interdisciplinary science requires lasting support to thrive. The Open Microscopy Environment (OME; Goldberg et al., 2005) offers a concrete example of the time scales and resources necessary to produce a lasting impact on data sharing culture, as their efforts towards building and maintaining support for the diversity of acquisition formats in microscopy imaging has required tens of millions in public funding over the past 20 years. Additional endeavours to build and maintain community standards, such as the Consortium for Quality Assessment and Reproducibility for Instruments & Images in Light Microscopy (QUAREP-LiMi) on the data producer side (Boehm et al., 2021) and AI4Life (Ouyang et al., 2022 preprint) on the data consumer side, will likely need equal levels of continued and dedicated support. Major commitments will also be needed to practically implement community-defined standards in academic infrastructures, convince commercial vendors to adopt them, and expedite data sharing and reuse. In this regard, university core facilities can play an essential role by facilitating the adoption of the FAIR principles across institutions or even countries, thereby driving alignment across the international bioimaging community (Schmidt et al., 2024). To enable this, however, significant and long-term resources will be needed to support the ecosystem of individuals involved in improving the experience for both producers and consumers.

Funding agencies are increasingly driving positive changes by mandating all bioimaging data generated with their support to be openly shared. Similarly, scientific publishers are playing an important role in enforcing deposition of all bioimage datasets in publicly accessible image archives prior to publication. However, these mandates can only be effective when supported by community-endorsed platforms for data sharing. Expecting immediate success is unreasonable, and thus strategic actions such as datathons must be taken to make bioimage data sharing the norm. Large-scale endeavours to survey the needs of the bioimage analysis community (Sivagurunathan et al., 2023) and build capacity for long-term commitments, including training and career progression pathways for data stewards, research software engineers and image analysts (Wright et al., 2024), are also valuable, but only form the very first steps toward facilitating effortless interactions between producers, consumers and everyone in between. Nevertheless, these developments promise a bright future as long as we keep in mind that there is so much more still to be done.
